# Acute bout of exercise induced prolonged muscle glucose transporter-4 translocation and delayed counter-regulatory hormone response in type 1 diabetes

**DOI:** 10.1371/journal.pone.0178505

**Published:** 2017-06-01

**Authors:** Koji Sato, Takeshi Nishijima, Takumi Yokokawa, Satoshi Fujita

**Affiliations:** 1 Faculty of Sport and Health Science, Ritsumeikan University, Shiga, Japan; 2 Graduate School of Human Development and Environment, Kobe University, Kobe, Japan; 3 Department of Health Promotion Sciences, Tokyo Metropolitan University, Tokyo, Japan; 4 Graduate School of Human and Environmental Studies, Kyoto University, Kyoto, Japan; Max Delbruck Centrum fur Molekulare Medizin Berlin Buch, GERMANY

## Abstract

Previous studies have demonstrated that an acute bout of aerobic exercise induces a subsequent delayed onset of hypoglycemia among patients with type 1 diabetes. However, the mechanisms of exercise-induced hypoglycemia in type 1 diabetes are still unclear. Streptozotocin (STZ) was injected to 6-week-old male Wistar rats, and three days after STZ injection, animals were randomly assigned into 2 groups: STZ with insulin only (STZ) and STZ with insulin and exercise (STZ+EX). Normal Wistar rats with exercise were used as control (CON+EX). Insulin was intraperitoneally injected (0.5 U/kg) to both STZ groups (−0.5 h), and a bout of aerobic exercise (15 m/min for 30 min) was conducted at euglycemic conditions (0 h). Blood was collected at 0, 1, 3, and 5 h after exercise from the carotid artery. While the blood glucose level was stable during the post-exercise period (0–5 h) in the STZ and CON+EX groups, it decreased significantly only in the STZ+EX group at 3 h. Plasma glucagon, adrenalin, and noradrenalin levels significantly increased at 1 h in the STZ group, whereas significant hormonal responses were observed at 5 h in the STZ+EX group. In skeletal muscle glucose metabolism-related pathway, the level of glucose transporter-4 (GLUT-4) translocation was significantly higher at 1 h in the CON and STZ groups. However, in the STZ+EX group, these activations were maintained by 5 h, indicating a sustained glucose metabolism in the STZ+EX group. A single bout of aerobic exercise induced a delayed onset of hypoglycemia in STZ-treated rats. A prolonged enhancement of GLUT-4 translocation and delayed counter-regulatory hormone responses may have contributed to the induction of hypoglycemia.

## Introduction

Exercise-induced hypoglycemia occurs during and after exercise in patients with type 1 diabetes mellitus (T1DM), and exercise-induced changes in blood glucose level or hemoglobin A_1c_ (HbA1c) complicate the management of diabetes. Exercise guideline for patients with T1DM by American Diabetes Association recommend insulin doses and a supplementary meal before exercise to prevent hypoglycemia; however, patients with T1DM who exercise have been reported to experience a delayed onset of hypoglycemia post-exercise, which occurs up to 12–14 h or even longer after the end of exercise [1, 2]. It is crucially important to prevent delayed onset of hypoglycemia in patients with T1DM for glycemic control. Although late-onset hypoglycemia has been suggested to occur by replenishing of the stored muscle glycogen and by a sustained increase in tissue sensitivity to insulin [3], its mechanism has not been explored.

For regulating glucose transporter-4 (GLUT-4), it is crucial that insulin binds to insulin receptor substrate 1 (IRS-1) and upregulates the activation of phosphoinositide 3-kinase (PI3 kinase) and phosphorylation of protein kinase B (Akt) in the skeletal muscle [4]. The enhancement of GLUT-4 translocation causes the increase in glucose uptake and decrease in blood glucose level. Hyperglycemic condition decreases GLUT-4 expression and its plasma membrane contents [5], and insulin deficiency induces abnormalities in the glucose metabolism-related signaling pathway in the skeletal muscle for patients with T1DM [6]. According to previous studies, rats with T1DM induced by streptozotocin (STZ) exhibit many changes in glucose metabolism, including reductions in GLUT-4 protein, PI3 kinase, and Akt phosphorylation in the skeletal muscles [7, 8].

Aerobic exercise generally induces enhancement of these glucose metabolism signals in the skeletal muscles. Hypoglycemia after exercise is often caused by increased glucose uptake and/or decreased glucose production [9]. In normal healthy individuals, exercise-induced increase in GLUT-4 translocation occurs immediately after and till 1 h in response to a single bout of exercise and decreases within 3 h post-exercise [10]. Moreover, blood glucose level by exercise in normal healthy individuals is generally maintained during exercise and early recovery period due to counter-regulatory systems and is attributed to maintain the glucose appearance and the glucose utilization rates [11]. However, many patients with T1DM demonstrate impaired response of glucagon and epinephrine to hypoglycemia implicating a possible defect in glucose production during hypoglycemia [1, 2]. Although the exercise causes increase in glucose output during exercise, it may still expose the patients to a higher risk of post-exercise hypoglycemia. Nevertheless, the mechanism of exercise-induced delayed onset of hypoglycemia in T1DM is still unclear.

Therefore, the aim of this study was to investigate the change in muscle glucose metabolism-related signaling and counter-regulatory hormones in response to exercise-induced hypoglycemia in T1DM model rat. We hypothesized that moderate aerobic exercise induces delayed increase in GLUT-4 translocation in T1DM model rats.

## Materials and methods

Ethical approval for this study was obtained from the Committee on Animal Care at the Ritsumeikan University. Male Wistar rats (5-week-old) were obtained from Charles River Japan Inc. (Kanagawa, Japan) and cared for according to *The Guiding Principles for the Care and Use of Animals*, based on the Declaration of Helsinki. These rats were maintained on a 12:12 h light–dark cycle and received *ad libitum* access to food and water.

**Fig 1:**
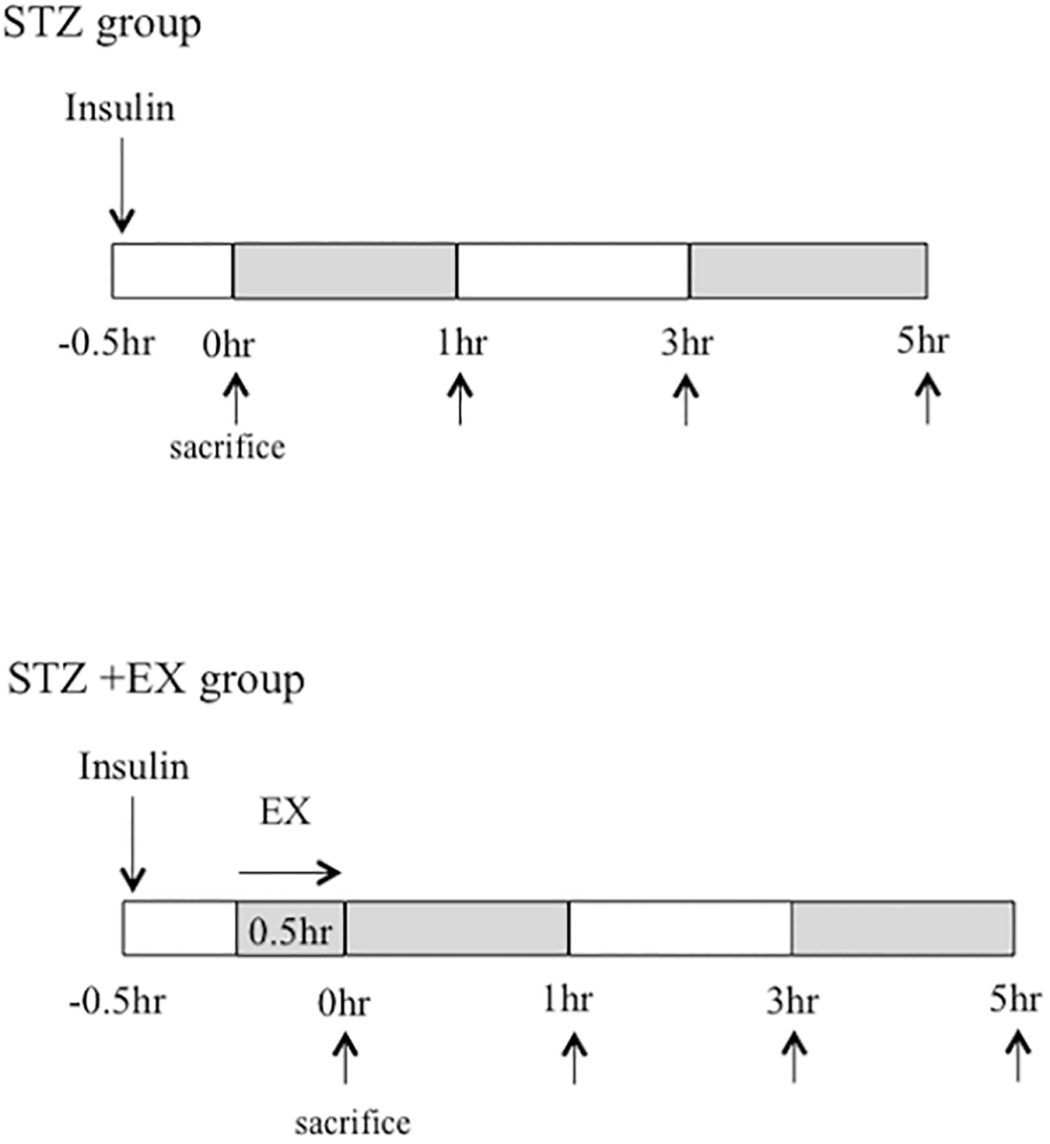
Schematic diagram of the experimental protocol. STZ, streptozotocin; EX, exercise. Insulin infusion was conducted before 30 min of exercise (−0.5 h) in both the STZ groups to start exercise at euglycemic condition.

At 6 weeks of age, a single dose of STZ (Sigma Chemical Co., St. Louis, MO, USA) was injected intraperitoneally to induce T1DM. STZ was dissolved in 0.1 M sodium citrate buffer (pH 4.5) and injected at a dosage of 55 mg/kg. Weight was checked before and after STZ injection. Three days after STZ injection, the fasting glucose level was determined in a blood sample collected from the tail vein. Diabetes was defined as fasting blood glucose levels greater than 349.2 mg/dL, as described in previous studies (Sato et al, 2009). The animals were assigned into two groups: STZ with insulin (STZ; n = 24) and STZ with insulin and exercise (STZ+EX; n = 24). Normal Wistar rats with exercise were used as a control (CON+EX; n = 24). The cannula was tunneled subcutaneously, exteriorized at the back of the neck, and connected to a dual channel swivel via a tethering system (Instech, Plymouth Meeting, PA). Inhalation anesthesia was used for blood glucose monitoring, and 3% isoflurane was used during cannulation. Two days after cannulation, fasting blood glucose level in STZ was approximately 180.8–399.6 mg/dL after overnight fasting (8 h). Next, insulin (Novolin R; Novo Nordisk Pharm Ltd., Tokyo, Japan) was intraperitoneally injected (0.5 U/kg) to both the STZ groups (−0.5 h) to confirm euglycemic condition, and a bout of aerobic exercise (15 m/min for 30 min with treadmill running, and without decline and electric shock) was conducted for the STZ+EX and CON+EX groups. The blood glucose level was monitored (Breeze 2; Bayer Health Care, Tokyo, Japan) at 1, 3, and 5 h after a single bout of exercise (Fig 1). Isoflurane (4%) with inhalation anesthesia was used for muscle sampling, and 5% isoflurane with inhalation anesthesia was applied to alleviate suffering and to euthanize the rats. Muscle samples were obtained at 0, 1, 3, and 5 h after completion of exercise (n = 6 for each time point). For each time points, heart and plantaris and soleus and gastrocnemius muscles were resected quickly, rinsed in ice-cold saline, and frozen in liquid nitrogen and stored at −80°C until use. The gastrocnemius muscle was used to evaluate glucose metabolism signaling phosphorylation and GLUT-4 translocation according to previous studies [12, 13, 14, 15].

### Immunoblot analysis

The muscle specimen was homogenized with 2 mM dithiothreitol (DTT), 20 mM Tris-HCl (pH 7.8), 300 mM NaCl, 2 mM ethylenediaminetetraacetic acid (EDTA), 0.2% sodium lauryl sulfate (SDS), 2% Nonidet P-40 (NP-40), 0.2% sodium deoxycholate, 0.5 mM phenylmethylsulfonyl fluoride (PMSF), 50 μg/ml aprotinin, and 1 μ and leupeptin. The homogenate was rotated gently for 30 min at 4°C and centrifuged at 12,000 ×*g* for 10 min at 4°C. The protein concentration of the supernatant was determined. Samples (30 μg protein) were denatured at 96°C for 7 min in Laemmli buffer. Western blot analysis was performed to detect GLUT-4 protein expression and Akt phosphorylation [12,13]. Each sample was separated on a 10% SDS–polyacrylamide gel and transferred to a polyvinylidene difluoride (PVDF; Millipore Corp., Billerica, MA, USA) membrane. Blocking buffer, which contained 5% skim milk in phosphate buffered saline with 0.1% Tween 20 (PBS-T), was used to treat the membrane for 24 h at 4°C. The membrane was probed with anti-GLUT4 (Merck Millipore, Darmstadt, Germany) anti-serine (Ser) 473-phosphorylated Akt or anti-Akt, antibody (Cell Signaling, Beverly, MA, USA); all antibodies were diluted to 1:1000 with the blocking buffer [14, 15]. The membrane was washed three times with PBS-T and incubated at room temperature for 1 h with a horseradish peroxidase (HRP)-conjugated secondary antibody, anti-rabbit immunoglobulin, which were diluted to 1:3000 in the blocking buffer. The membrane was washed three times with PBS-T. Finally, GLUT4, phosphorylated Akt, and total Akt proteins were detected using an enhanced chemiluminescence system (ECL Prime; GE Healthcare Biosciences, Piscataway, NJ, USA) and visualized using an Image Quant LAS 4000 (GE Healthcare Biosciences).

### Preparation of the cytosolic and plasma membrane protein fractions

To assess GLUT-4 translocation, two membrane fractions were prepared as previously described [14]. To prepare the crude membrane, muscles were scraped into buffer A (1 mM EDTA, 20 mM Tris [pH 7.4], 0.25 M sucrose, 0.25 mM EGTA, 1 mM DTT, 25 mM sodium pyrophosphate, 50 mM NaF, and 40 mM β-glycerophosphate). The resulting homogenates were clarified by centrifugation at 500 ×*g* at 4°C for 15 min. The supernatant was centrifuged at 100,000 ×*g* for 1 h. The enriched GLUT-4 membrane fraction was produced by blending the muscles five times for 20 s in Tris buffer containing 0.25 mM PMSF and 1.4 M sucrose. The homogenate was centrifuged at 1,500 ×*g* at 4°C for 10 min. EDTA (1 mM) was added to the postnuclear supernatant, and the solution was prepared for density centrifugation by overlaying the postnuclear supernatant with Tris buffer containing 1.2 and 0.8 M sucrose. The enriched GLUT-4 membrane fraction was harvested in the 0.8/1.2 M sucrose interphase. Two hundred microliters of the harvested interphase sample was resuspended in buffer A. For preparation of the cytosolic protein fraction, cells were solubilized for 1 h in buffer B (20 mM Tris [pH 7.4], 2 mM CaCl_2_, 1 mM EDTA, 70 mM KCl, 3 mM magnesium acetate). The homogenate was centrifuged 1,500 ×*g* at 4°C for 10 min., and the supernatant was centrifuged for 1 h at 100,000 ×*g*. The supernatant was used as the cytosolic fraction. GLUT-4 protein levels were measured in both the membrane and cytosolic fractions. Translocation was determined as the difference in protein levels between the cytosol and membrane fractions [14].

### Glucose-6-phosphate (G-6-P) analysis

G-6-P level was detected using the Glucose-6-phosphate fluorometric assay kit (Cayman, Ann Arbor, MI, USA). The assay was conducted according to manufacturer’s protocol. Measurement of fluorescence were conducted by Fluoroskan Ascent (Thermo Scientific, Waltham, MA, USA) with an excitation wavelength of 530–540 nm and an emission wavelength of 585–595 nm.

### Sandwich-enzyme immunoassay (EIA)

The levels of adrenaline, noradrenaline (Cusabio Biotech Co., Baltimore, MD, USA), insulin (Mercodia, Uppsala, Sweden), glucagon (R&D systems, Inc, Minneapolis, MN, USA), and adropin (Phoenix Pharmaceuticals, Inc. Burlingame, CA, USA) in the plasma were determined using a sandwich-EIA kit. All techniques and materials used in these analyses were in accordance with the manufacturer’s protocol. The immobilized antibodies were polyclonal antibodies against adrenaline, noradrenaline, glucagon, and adropin, whereas the secondary horseradish peroxidase-coupled antibody was monoclonal. Optical density at 420 was determined on a microplate reader (xMark; Bio-Rad, Hercules, CA, USA).

### Statistical analysis

All values are expressed as means ± SE. Statistical evaluations for the parameters were performed using two-way ANOVA for repeated measures (time × group). A post-hoc comparison test was used to correct for multiple comparisons (Bonferroni test) when analyses revealed significant differences. For ANOVA, *P* < 0.01 was considered significant.

## Results

### Plasma glucose level

**Fig 2:**
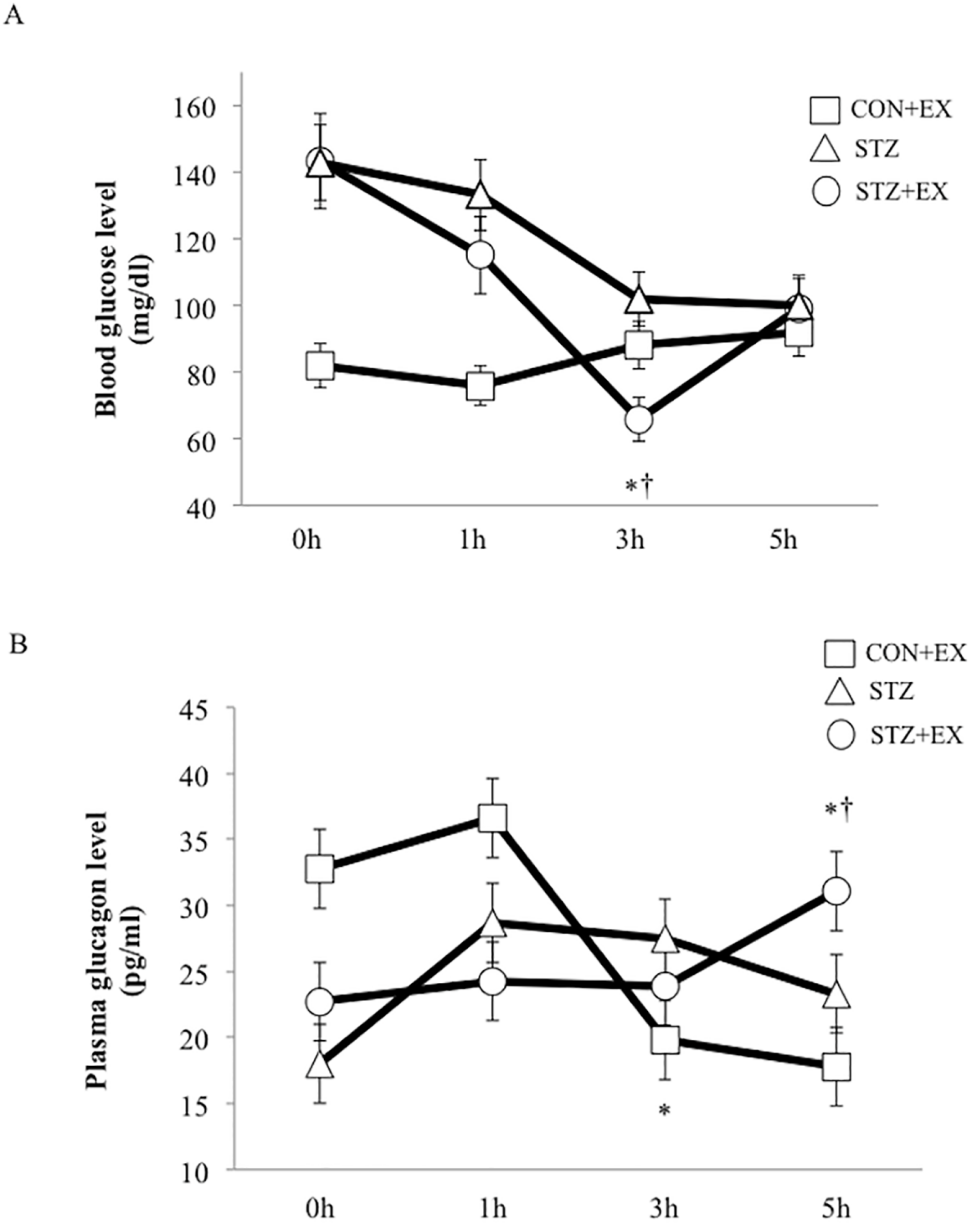
**The effect of a single bout of exercise on plasma glucose (A) and glucagon (B) levels.** * P < 0.05 vs. 0 h, † P < 0.05 vs. STZ. Values are represented as mean ± SE.

Blood glucose level decreased significantly at 3 h after exercise in the STZ+EX group compared to other groups, whereas blood glucose in the STZ group decreased gradually, but the decrease was not statistically significant (Fig 2A).

### Glucagon, catecholamine, and insulin levels

**Fig 3:**
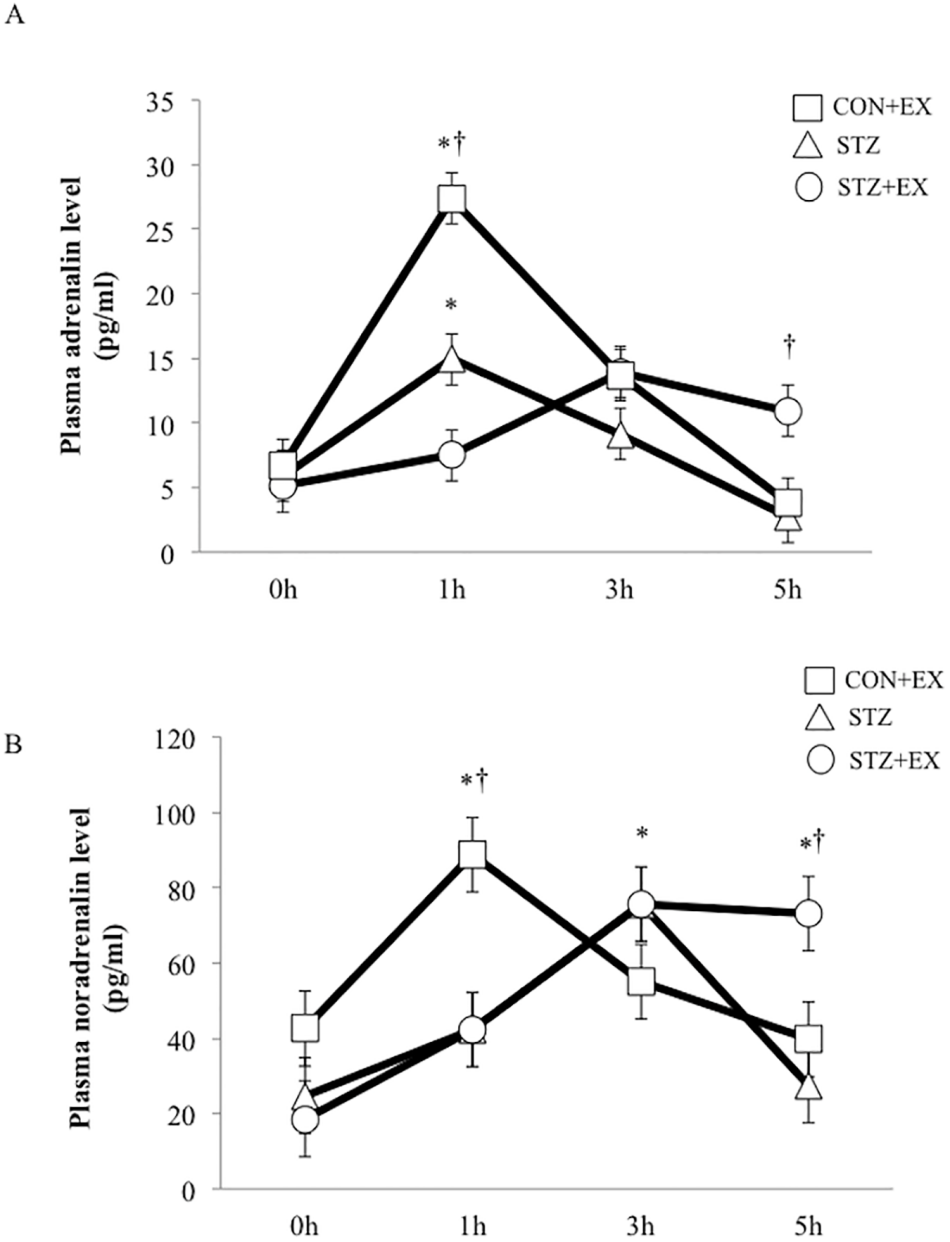
**The effect of a single bout of exercise on plasma adrenalin (A) and noradrenalin (B) levels.** * P < 0.05 vs. 0 h, † P < 0.05 vs. STZ. Values are represented as mean ± SE.

**Fig 4:**
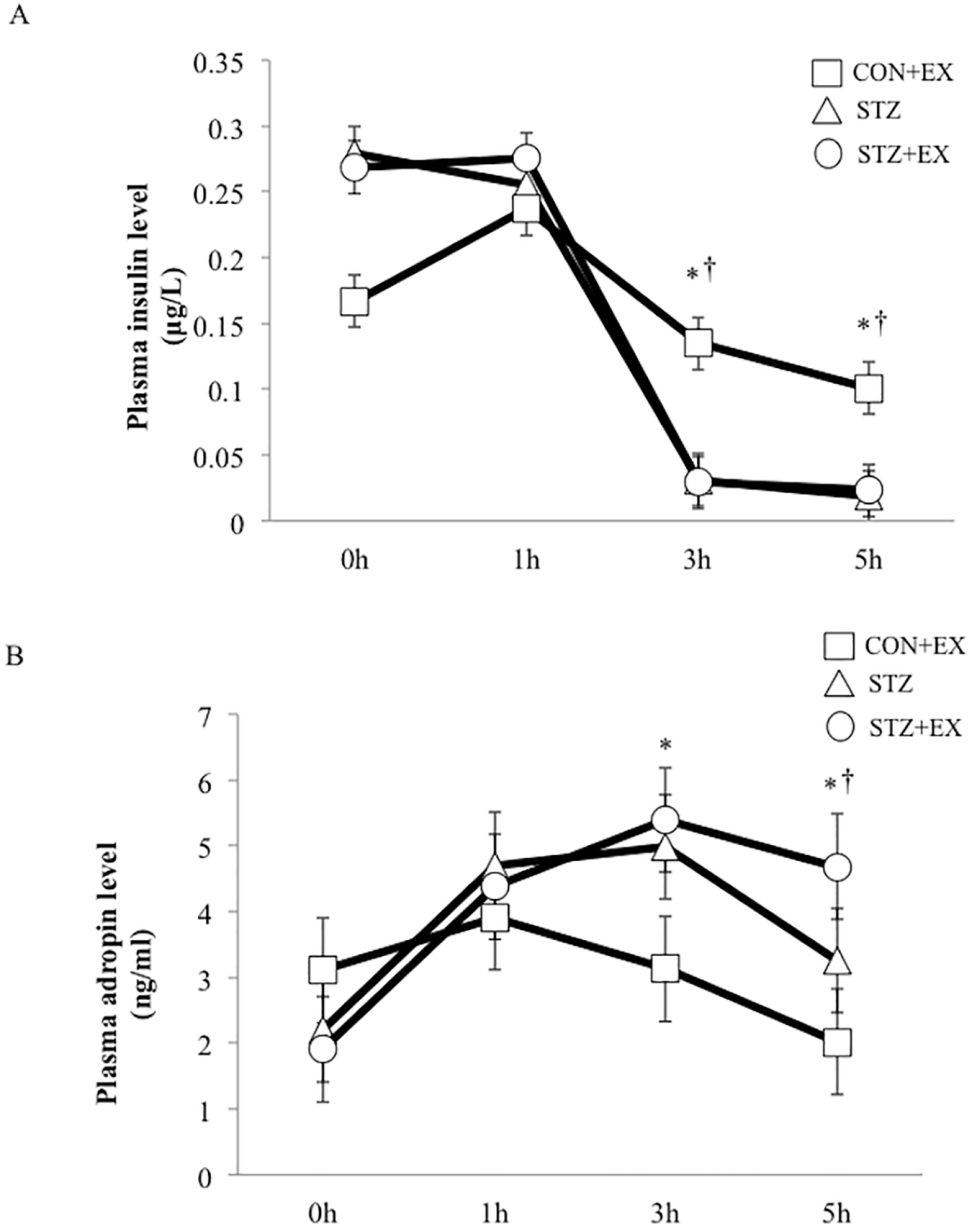
**The effect of a single bout of exercise on plasma insulin (A) and adropin (B) levels.** * P < 0.05 vs. 0 h, † P < 0.05 vs. STZ. Values are represented as mean ± SE.

Plasma glucagon level increased slightly at 1 h after exercise in the CON+EX group. Plasma glucagon level did not change significantly in the STZ+EX group at 1 h after exercise, but it increased significantly at 5 h after exercise compared to other groups (P < 0.01; Fig 2B). Similarly, significant elevation of adrenaline was observed in the CON+EX group at 1 h after exercise, but plasma adrenalin level increased significantly only at 5 h after exercise in the STZ+EX group (Fig 3A). Moreover, noradrenalin level increased significantly at 1 h after exercise in the CON+EX group, and prolonged increases in plasma noradrenalin level were observed between 3–5 h after exercise in the STZ+EX group (Fig 3B), although noradrenalin level decreased at 5 h in the STZ and CON+EX groups. Plasma insulin level was slightly higher in both the insulin-treated STZ groups (STZ and STX+EX); however, no significant changes were observed at 1, 3, and 5 h between the STZ and STZ+EX groups (Fig 4A).

### Adropin level

No significant difference in plasma adropin concentration was observed between groups at baseline. In all groups, plasma adropin level increased after aerobic exercise. However, the adropin concentration was significantly higher only in the STZ+EX group compared with that of other groups at 5 h after exercise (Fig 4B).

### Glucose metabolism-related signaling

**Fig 5:**
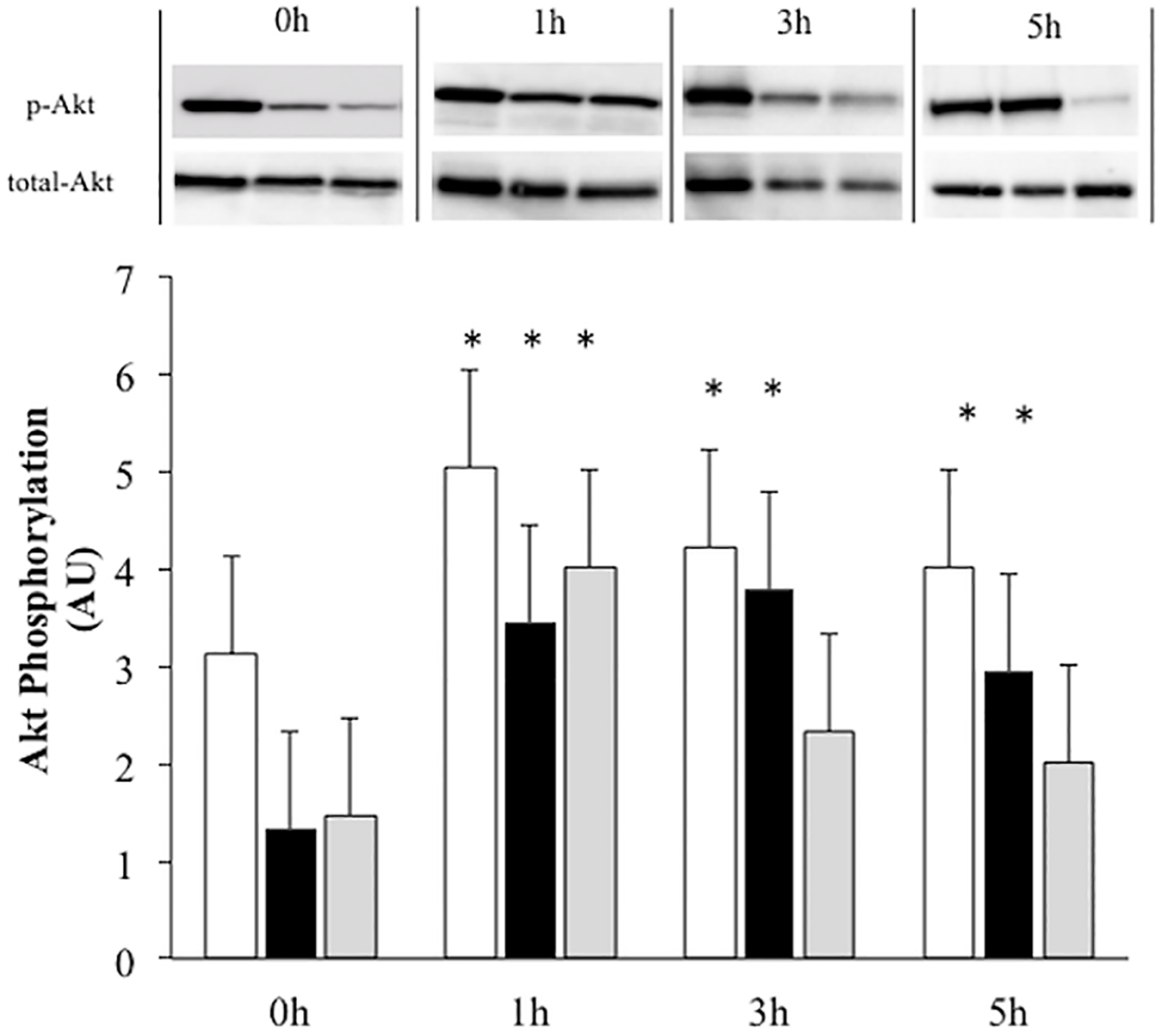
The effect of a single bout of exercise on Akt phosphorylation Ser^473^ relative to total protein. White bar, CON+EX; Black bar, STZ+EX; Gray bar, STZ. * P < 0.05 vs. 0 h. Values are represented as mean ± SE. The gels were electrophoresed under the same experimental conditions.

Akt phosphorylation increased significantly after exercise in both the CON and STZ rats at 1 h, and in the CON + EX group, Akt phosphorylation decreased gradually till 5 h after exercise. Moreover, in the STZ+EX group, prolonged increase in phosphorylation was observed at 1–5 h after the exercise (Fig 5).

### GLUT-4 translocation

**Fig 6:**
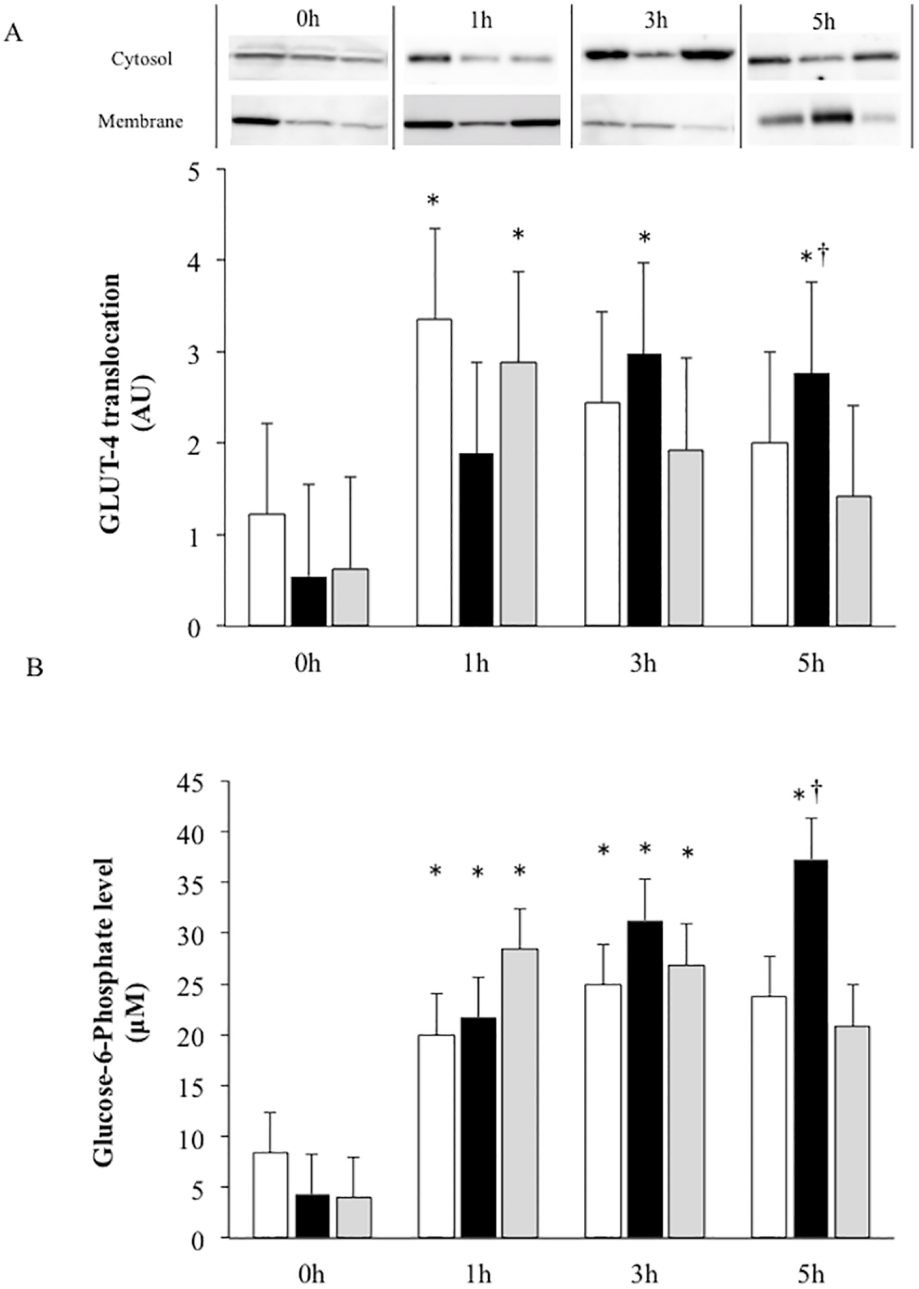
**The effect of a single bout of exercise on level of GLUT-4 translocation (A) and glucose-6-phosphate level (B).** White bar, CON+EX; Black bar, STZ+EX; Gray bar, STZ. * P < 0.05 vs. 0 h, † P < 0.05 vs. STZ. Values are represented as mean ± SE. The gels were electrophoresed under the same experimental conditions.

GLUT-4 translocation level pronounced at 1 h after the treatment in the CON+EX and STZ groups and gradually decreased at 5 h after exercise, whereas GLUT-4 translocation pronounced at 3 h, and a prolonged increase was observed till 5 h after exercise (Fig 6A).

### G-6-P level

As an index of glucose utilization, G-6-P level was measured in the skeletal muscles. In all groups, G-6-P significantly increased at 1 and 3 h after exercise. Additionally, muscle G-6-P level increased significantly till 5 h after exercise only in the STZ+EX group, although G-6-P level decreased to a non-significant level by 5 h after exercise in the STZ and CON+EX groups (Fig 6B).

## Discussion

In the present study, we firstly revealed that a single bout of moderate intensity exercise for T1DM model rats induced an antecedent decrease in blood glucose level at 3 h after exercise without changes in the insulin level. One of the mechanisms may be a prolonged enhancement of GLUT-4 translocation in the muscles. In addition, exercise-induced increase in plasma adropin level was prolonged after exercise. Consequently, prolonged enhancement of GLUT-4 translocation in muscle and delayed onset of counter-regulatory response may have contributed to a delayed onset of hypoglycemia in T1DM.

Although promoting physical fitness and health is vital for patients with diabetes, exercise may be the most frequent behavior that causes dysglycemia in patients with T1DM. Exercise-induced hypoglycemia causes dangerous implications as well as discourages individuals wishing to exercise [16]. Previous studies have demonstrated that blood glucose level decreases 6 h after exercise in patients with T1DM [11, 17]. Moreover, 75 min of treadmill walking for children with T1DM in the late afternoon is associated with a high frequency of hypoglycemia during nighttime sleep [18]. However, the time-course change in muscle glucose metabolism, which is induced by a single bout of exercise, in T1DM has not been explored. In normal healthy individuals, acute exercise-induced enhancement of GLUT-4 translocation occurs immediately after 1 h and gradually ceases to baseline level within 3 h post-exercise, and the blood glucose levels are usually maintained after exercise in normal healthy individuals [10].

In the present study, GLUT-4 translocation pronounced at 1 h after exercise and decreased within 5 h in normal healthy rats, and blood glucose levels were maintained after exercise. On the contrary, in the STZ+EX group, GLUT-4 translocation level pronounced at 3 h and was maintained till 5 h in T1DM, whereas the blood glucose level rapidly decreased at 3 h after exercise. The present study demonstrated a time-course change in exercise-induced GLUT-4 translocation level in T1DM; however, the reasons that delayed the enhancement of GLUT-4 translocation in T1DM are still unclear. Moderate aerobic exercise (15 m/min treadmill running for 30 min) was conducted under euglycemic conditions (118.8–138.6 mg/dL) in the present study, and insulin was injected 30 min before the exercise in the STZ+EX group. In the STZ group, GLUT-4 translocation level pronounced within 1.5 h after insulin injection in the present study (Fig 1). Moreover, G-6-P level in muscle was also measured in the present study as an index of glucose uptake; the levels pronounced at 5 h after exercise only in the STZ+EX group with T1DM, whereas a significant increase was observed up to 3 h in the CON and STZ+EX groups.

Circulating peptide hormone adropin, which plays a role in energy homeostasis level was measured in the present study. Previous studies have reported that adropin activates pyruvate dehydrogenase (PDH), a rate-limiting enzyme in glucose oxidation, and downregulates PDH kinase-4, which inhibits PDH [19, 20]. Moreover, transgenic overexpression of adropin or treatment with adropin improves glucose clearance and reduces fasting insulin levels in diet-induced obese mice [21]. Therefore, adropin has the potential to enhance glucose utilization. We observed that circulating adropin level increased slightly at 1 h post-treatment in all the groups in the present study. Although the levels gradually decreased at 5 h after exercise in the STZ and CON groups, the adropin level was maintained at higher level in the STZ+EX group. The significant increase in adropin level at 5 h after exercise may have contributed to a prolonged increase in GLUT-4 translocation level in T1DM; however, further study is needed to clarify the effect of adropin on GLUT-4 translocation using adropin-knockout mice.

Although it is feasible to assume that the delayed onset of hypoglycemia was triggered by the prolonged translocation and increased glucose metabolism in skeletal muscles, the mechanism by which the sympathoadrenal response to hypoglycemia was delayed in the STX+EX group remains to be elucidated. Glycemic thresholds for autonomic and symptomatic responses to hypoglycemia are shifted to lower plasma glucose concentrations in intensively treated patients with T1DM [22, 23] exposing the patients to hypoglycemia unawareness and the resulting severe hypoglycemia [24]. Antecedent hypoglycemia is assumed to mediate these threshold shifts from the intensive therapy [25]. Previous studies have indicated that antecedent hypoglycemia reduces the counter-regulatory response to subsequent hypoglycemia [26]. However, in the current study, animals were injected with STZ three days prior to the experiment and were never exposed to hypoglycemia or exogenous insulin until the study day. Therefore, the blunted counter-regulatory response observed in the STZ+EX group cannot be attributed to antecedent hypoglycemia or repetitive insulin treatment. Further studies are warranted to investigate the effect of prior exercise on delayed courter-regulatory response in patients with T1DM.

In summary, to the best of our knowledge, we are the first to report that a single bout of moderate-intensity exercise induces prolonged increase in muscle GLUT-4 translocation and that counter-regulatory response is blunted in response to exercise compared with normal healthy rats with exercise and T1DM model rats without exercise. Therefore, based on the results of this study, we recommend that insulin dose should be administered before a meal and before exercising and carbohydrates should be consumed before bedtime in patients with T1DM who exercise for exercise-induced prolonged enhancement of glucose metabolism signaling at 3 h to 5 h after exercise. However, further studies are required to investigate longer-time monitoring periods of 8, 12, and 24 h after exercise and to determine exercise-induced changes in muscle and liver glycogen signaling.

## Supporting information

S1 Table Raw data of present study. (XLSX)

S1 Fig Certification which native speaker checked English language in the manuscript. (PDF)
