# Supplementary material for: Acute bout of exercise induced prolonged muscle glucose transporter-4 translocation and delayed counter-regulatory hormone response in type 1 diabetes
Source: PLoS One. 2017 Jun 1;12(6):e0178505. doi: 10.1371/journal.pone.0178505 (PMC5453522; doi:10.1371/journal.pone.0178505)
Supplement: S1 Fig — (PDF) [file pone.0178505.s002.pdf]

CERTIFICATE OF ENGLISH EDITING

This document certifies that the paper listed below has been edited to ensure that the language is clear and free of errors. The edit was performed by professional editors at Editage, a division of Cactus Communications.The intent of the author's message was not altered in any way during the editing process. The quality of the edit has been guaranteed, with the assumption that our suggested changes have been accepted and have not been further altered without the knowledge of our editors.

Title Of The Paper

Acute bout of exercise induced prolonged muscle glucose transporter-4 translocation and delayed counter-regulatory hormone response in type 1 diabetes

Authors

Koji Sato

Job Code

XJCPO_2_2

Signature

Vikas Narang

Vikas Narang,
Vice President, Author Services, Editage

Date of Issue
May 15, 2017

Editage, a brand of Cactus Communications, offers professional English language editing and publication support services to authors engaged in over 500 areas of research. Through its community of experienced editors, which includes doctors, engineers, published scientists, and researchers with peer review experience, Editage has successfully helped authors get published in internationally reputed journals. Authors who work with Editage are guaranteed excellent language quality and timely delivery.

Contact Editage

Worldwide
request@editage.com
+1 877-334-8243
www.editage.com

Japan
submissions@editage.com
+81 03-6868-3348
www.editage.jp

Korea
submit-korea@editage.com
1544-9241
www.editage.co.kr

China
fabiao@editage.cn
400-005-6055
www.editage.cn

Brazil
inquiry.brazil@editage.com
0800-892-20-97
www.editage.com.br

Taiwan
submitjobs@editage.com
02 2657 0306
www.editage.com.tw
